# Supplementary material for: CXCR2 Antagonist RIST4721 Acts as a Potent Chemotaxis Inhibitor of Mature Neutrophils Derived from Ex Vivo-Cultured Mouse Bone Marrow
Source: Biomedicines. 2023 Feb 7;11(2):479. doi: 10.3390/biomedicines11020479 (PMC9953560; doi:10.3390/biomedicines11020479)
Supplement: Supplementary file 1 [file biomedicines-11-00479-s001.zip › biomedicines-2172562-supplementary.pptx]

## Slide 1
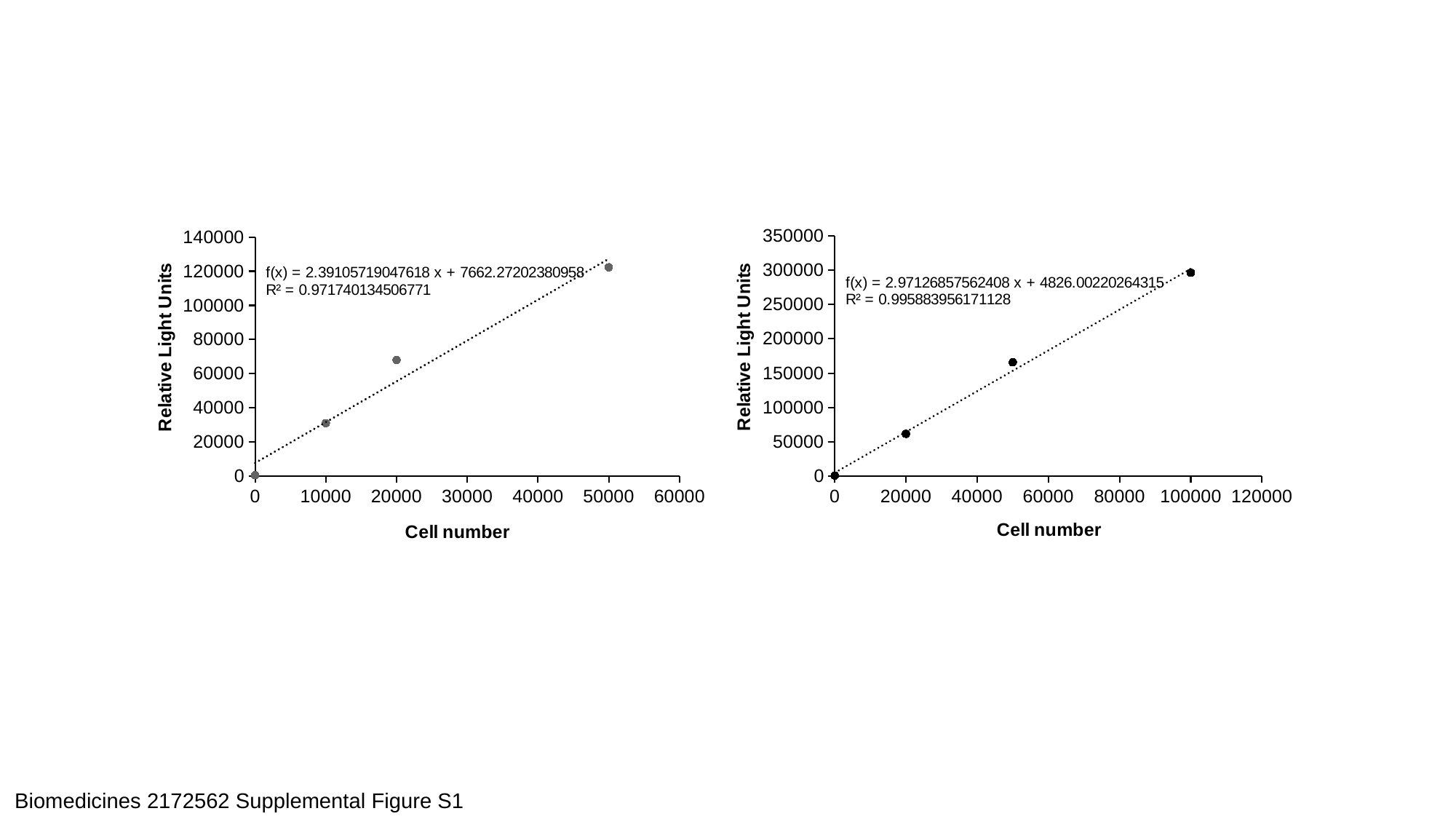

### Chart
| Category | |
|---|---|
### Chart
| Category | |
|---|---|Biomedicines 2172562 Supplemental Figure S1

## Slide 2
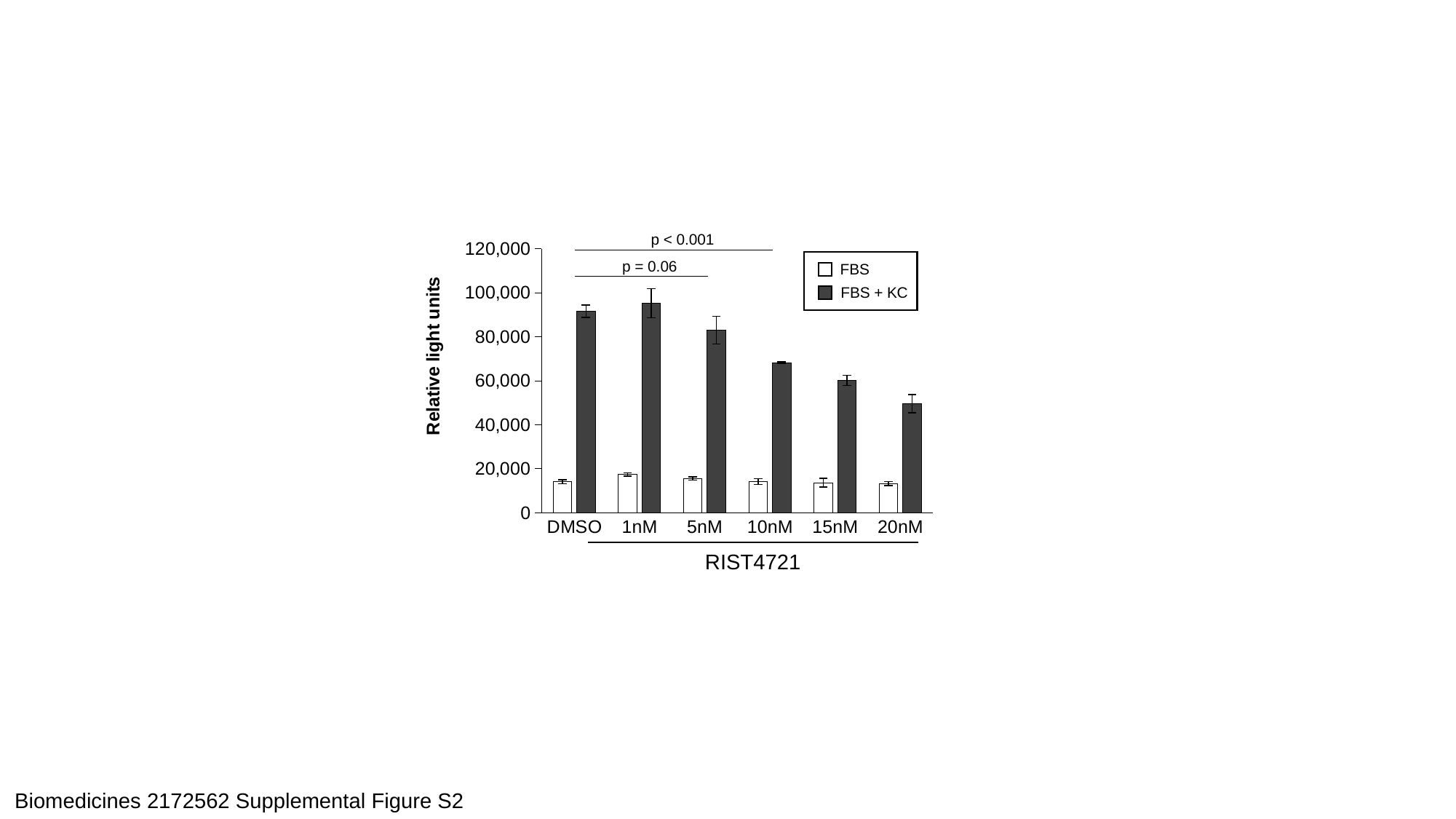

p < 0.001
### Chart
| Category | FBS | FBS+KC |
|---|---|---|
| DMSO | 14128.67 | 91653.33666666667 |
| 1nM | 17393.00333333333 | 95254.00333333334 |
| 5nM | 15579.003333333334 | 83043.33666666667 |
| 10nM | 14143.003333333334 | 68298.33666666667 |
| 15nM | 13679.67 | 60215.33666666667 |
| 20nM | 13328.336666666668 | 49593.670000000006 |p = 0.06
FBS
FBS + KC
RIST4721
Biomedicines 2172562 Supplemental Figure S2
